# Supplementary material for: Facultative dosage compensation of developmental genes on autosomes in Drosophila and mouse embryonic stem cells
Source: Nat Commun. 2018 Sep 7;9:3626. doi: 10.1038/s41467-018-05642-2 (PMC6128902; doi:10.1038/s41467-018-05642-2)
Supplement: Supplementary file 1 — Description of Additional Supplementary Files [file 41467_2018_5642_MOESM1_ESM.pdf]

Description of Additional Supplementary Files

File Name: Supplementary Data 1
Description: Comprehensive overview of all ChIP-seq, FLASH and RNA-seq datasets generated in this study.

File Name: Supplementary Data 2
Description: a) Complete list of Drosophila MSL2tg-bound genes (MACS peak within 200 bp of a TSS excluding HAS peaks) b) Same analysis, but only a single gene overlap allowed c)-e) Genelist of TSS peaks associated with Cluster 1, 2 and 3 (Figure 2).

File Name: Supplementary Data 3
Description: DEseq output of differentially expressed genes in Msl2Δ mESCs in Serum condition (FDR < 0.05).

File Name: Supplementary Data 4
Description: DEseq output of differentially expressed genes in Msl2Δ mESCs in 2i condition (FDR < 0.05).

File Name: Supplementary Data 5
Description: Individual datapoints of barplots presented in this study.
