# Supplementary material for: Facultative dosage compensation of developmental genes on autosomes in Drosophila and mouse embryonic stem cells
Source: Nat Commun. 2018 Sep 7;9:3626. doi: 10.1038/s41467-018-05642-2 (PMC6128902; doi:10.1038/s41467-018-05642-2)
Supplement: Supplementary file 3 — Supplementary Information [file 41467_2018_5642_MOESM3_ESM.pdf]

Facultative dosage compensation of developmental genes on autosomes in Drosophila and mouse embryonic stem cells

Valsecchi et al.

Supplementary Information

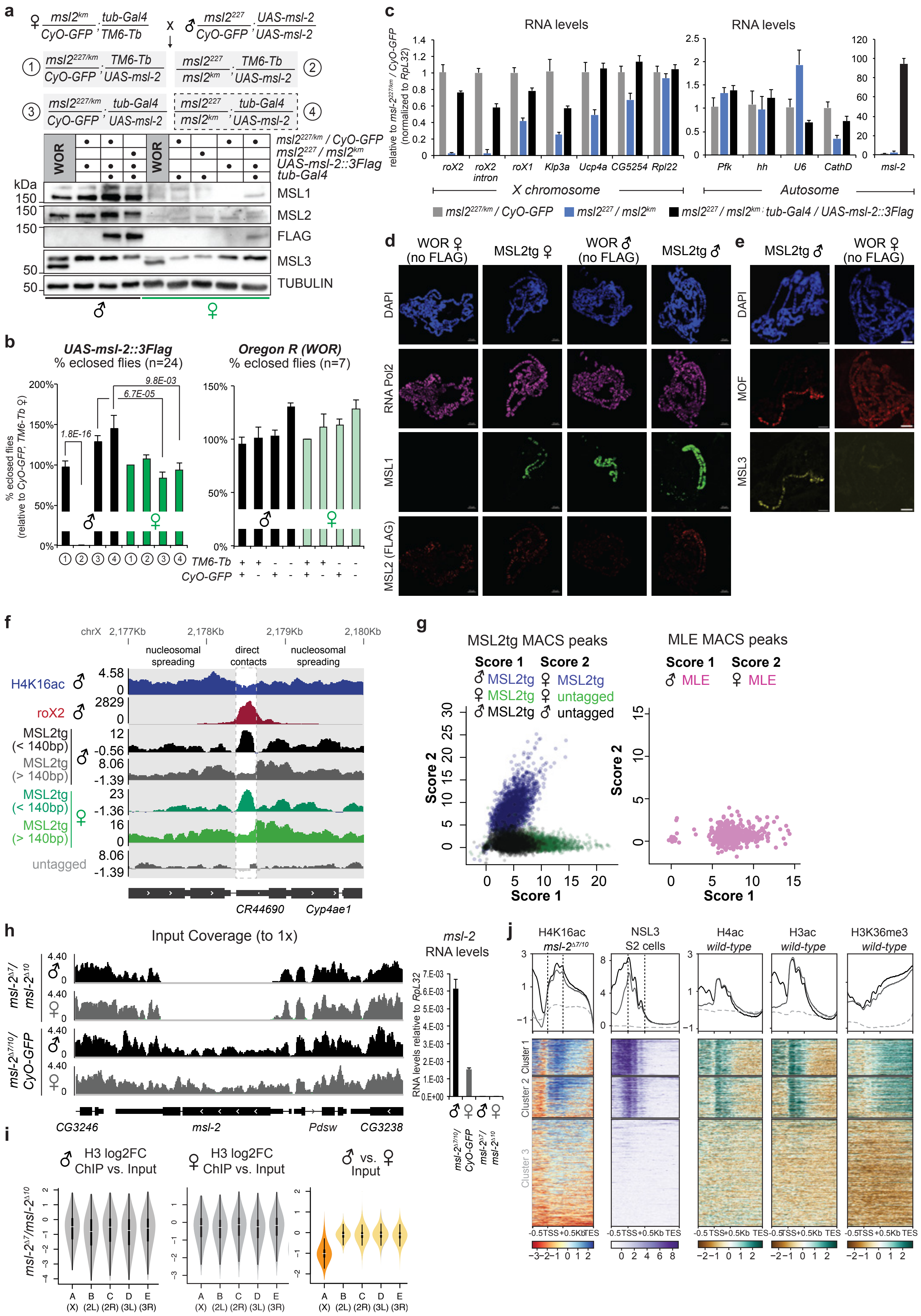
a
msl2km / CyO-GFP ; tub-Gal4 / TM6-Tb
x
msl2227 / CyO-GFP ; UAS-msl-2 / UAS-msl-2
1 msl2227/km / CyO-GFP ; TM6-Tb / UAS-msl-2
2 msl2227 / msl2km ; TM6-Tb / UAS-msl-2
3 msl2227/km / CyO-GFP ; tub-Gal4 / UAS-msl-2
4 msl2227 / msl2km ; tub-Gal4 / UAS-msl-2
WOR
msl2227/km / CyO-GFP
msl2227 / msl2km
UAS-msl-2::3Flag
tub-Gal4
kDa
150
MSL1
MSL2
FLAG
MSL3
50
TUBULIN
b
UAS-msl-2::3Flag
% eclosed flies (n=24)
Oregon R (WOR)
% eclosed flies (n=7)
% eclosed flies (relative to CyO-GFP, TM6-Tb ♀)
1.8E-16
6.7E-05
9.8E-03
200%
150%
100%
50%
0%
TM6-Tb
CyO-GFP
c
RNA levels
relative to msl-2227/km / CyO-GFP (normalized to RpL32)
roX2
roX2 intron
roX1
Klp3a
Ucp4a
CG5254
Rpl22
Pfk
hh
U6
CathD
msl-2
X chromosome
Autosome
msl2227/km / CyO-GFP
msl2227 / msl2km
msl2227 / msl2km ; tub-Gal4 / UAS-msl-2::3Flag
d
WOR ♀ (no FLAG)
MSL2tg ♀
WOR ♂ (no FLAG)
MSL2tg ♂
DAPI
RNA Pol2
MSL1
MSL2 (FLAG)
e
MSL2tg ♂
WOR ♀ (no FLAG)
DAPI
MOF
MSL3
f
chrX 2,177Kb 2,178Kb 2,179Kb 2,180Kb
nucleosomal spreading
direct contacts
nucleosomal spreading
H4K16ac ♂
roX2 ♂
MSL2tg (< 140bp)
MSL2tg (> 140bp)
untagged
CR44690
Cyp4ae1
g
MSL2tg MACS peaks
Score 1 Score 2
♂ MSL2tg ♀ MSL2tg
♀ MSL2tg ♀ untagged
♂ MSL2tg ♂ untagged
MLE MACS peaks
Score 1 Score 2
♂ MLE ♀ MLE
Score 2
Score 1
h
Input Coverage (to 1x)
msl-2Δ7/ msl-2Δ10
msl-2Δ7/10/ CyO-GFP
CG3246
msl-2
Pdsw
CG3238
msl-2 RNA levels
RNA levels relative to RpL32
i
♂ H3 log2FC ChIP vs. Input
♀ H3 log2FC ChIP vs. Input
♂ vs. ♀ Input
msl-2Δ7/msl-2Δ10
A (X) B (2L) C (2R) D (3L) E (3R)
j
H4K16ac msl-2Δ7/10
NSL3 S2 cells
H4ac wild-type
H3ac wild-type
H3K36me3 wild-type
Cluster 1
Cluster 2
Cluster 3
-0.5TSS +0.5Kb TES

Supplementary Figure 1

a) Characterization of transgenic *UAS-msl-2::3Flag* flies. The scheme indicates the crossing strategy and progeny obtained. Western blots from heads show *tub-Gal4* driven *UAS-msl-2::3Flag* protein expression levels nearly identical to the wild-type control male flies. In female heads, ectopic MSL2tg does not accumulate to the same levels as in males.

b) Progeny was counted from the offspring of crosses (24 vials) and expressed as a % of the *CyO, Act5C-GFP*, *TM6, Tb* female progeny. The numbers refer to genotypes stated in Supplementary Fig. 1a. Error bars represent the SD, P-values were calculated using a one-tailed t-test. Male lethality in *msl-2*^{227}*/msl-2*^{km} is fully rescued by ectopic expression of *UAS-msl-2::3Flag* with *tub-Gal4* at 25°C. Note the effect of the *CyO, Act5C-GFP* and *TM6, Tb* balancer chromosomes on viability of wild-type male and female flies (right panel). Ectopic expression of MSL2tg in females causes around 30% reduction in viability with a mildly delayed eclosion rate compared to controls (data not shown).

c) Real-time RT-qPCR analyses of the indicated genes from early male L3 larvae. The RNA level of each gene is expressed relative to the heterozygous *CyO, Act5C-GFP* progeny while normalizing to *RpL32*. The bar plot represents the average of 4 independently collected samples with error bars indicating the SEM.

d) Polytene squashes of male and female wild-type Oregon R (WOR) and MSL2tg lines (*msl-2*^{227} */ msl-2*^{km}*; tub-Gal4 / UAS-msl-2::3Flag)*. Squashes were immunostained with RNA Pol2, MSL1 and FLAG antibodies, respectively. Scale bar = 10 µm.

e) As in d) but staining for MOF and MSL3. Note that the contrast setting of the female WOR squash was set different compared with males to be able to visualize autosomal MOF staining within the NSL complex. Scale bar = 10 µm.

f) Genome browser snapshot of a selected HAS on the X chromosome illustrating the difference between small and large reads recovered in MNase-fragmented MSL2tg ChIP-seq. Coverage tracks were generated using deeptools bamCompare and plotted in IGV.

g) Enrichment scores on the merged list of all called MSL2tg peaks (left) or MLE peaks (right) were calculated using deeptools multiBigwigSummary and plotted in R. Male and female peaks are highly correlated in MSL2tg ChIPs (blue dots). No

enrichment can be detected in each corresponding untagged control. ChIP peaks for MLE can be only detected in males, but not females.

h) Characterization of the *msl-2*$^{\Delta 7}$ and *msl-2*$^{\Delta 10}$ CRISPR deletion alleles created in this study. The two alleles were independently obtained, but are identical in molecular nature, as assayed by DNA sequencing of PCR amplicons from the *msl-2* locus (data not shown). The read coverage of the "input" samples (H4K16ac ChIP-seq experiment) over the *msl-2* locus shows the absence of reads from the gene in *msl-2*$^{\Delta 7}$ / *msl-2*$^{\Delta 10}$ transheterozygous lines. Quantification of RNA levels by real-time qRT-PCR in early L3 larvae indicates the absence of any *msl-2* RNA expression normalized to *RpL32*. The barplot represents the average of 4 independently collected samples with error bars indicating the SEM.

i) Violin plots showing the distribution of enrichment scores per 1 kb bin on each chromosomal arm for the H3 ChIP performed in parallel to the H4K16ac ChIP shown in Figure 1. Scores were calculated using deeptools multiBigwigSummary and plotted in R. For the Inputs, the analyses were performed on log2FC Input (male) / Input (female) coverage files.

j) Heatmaps, where 3 unsupervised k-Means clusters were generated based on the H4K16ac ChIP-seq profiles in *msl-2*$^{\Delta 7}$ / *msl-2*$^{\Delta 10}$ mutant males. They were sorted according to the enrichment intensity within each cluster. The NSL3 ChIP from S2 cells[1], H4ac, H3ac and H3K36me3 ChIP (this study) from L3 larvae was plotted on the same regions keeping the order according to the clustering of the H4K16ac ChIP-seq.

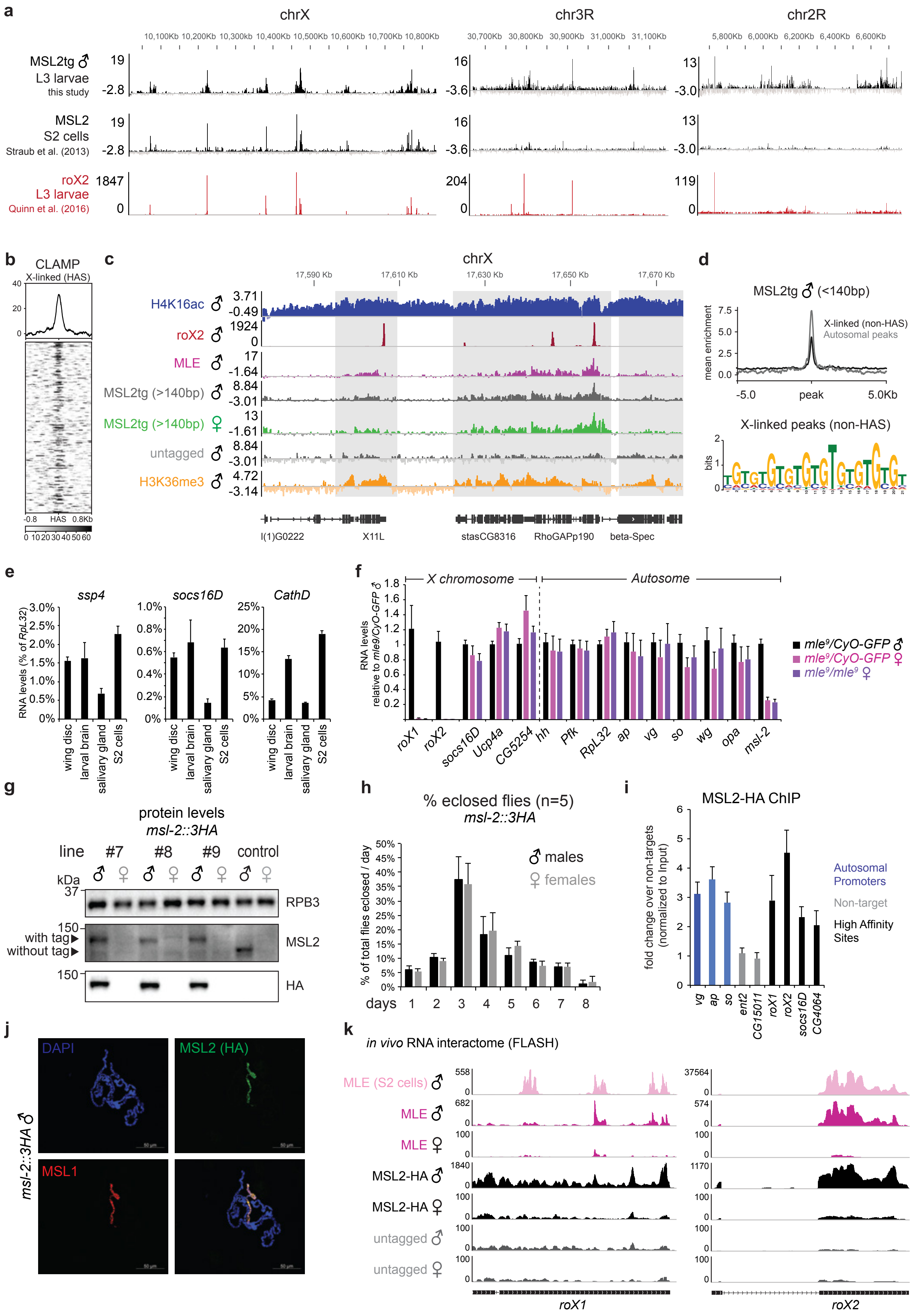

a
chrX
chr3R
chr2R
10,100Kb 10,200Kb 10,300Kb 10,400Kb 10,500Kb 10,600Kb 10,700Kb 10,800Kb
30,700Kb 30,800Kb 30,900Kb 31,000Kb 31,100Kb
5,800Kb 6,000Kb 6,200Kb 6,400Kb 6,600Kb
MSL2tg ♂
L3 larvae
this study
MSL2
S2 cells
Straub et al. (2013)
roX2
L3 larvae
Quinn et al. (2016)
b
CLAMP
X-linked (HAS)
-0.8 HAS 0.8Kb
c
chrX
17,590 Kb 17,610 Kb 17,630 Kb 17,650 Kb 17,670 Kb
H4K16ac ♂
roX2 ♂
MLE ♂
MSL2tg (>140bp) ♂
MSL2tg (>140bp) ♀
untagged ♂
H3K36me3 ♂
l(1)G0222
X11L
stasCG8316
RhoGAPp190
beta-Spec
d
MSL2tg ♂ (<140bp)
mean enrichment
X-linked (non-HAS)
Autosomal peaks
-5.0 peak 5.0Kb
X-linked peaks (non-HAS)
bits
e
ssp4
socs16D
CathD
RNA levels (% of RpL32)
wing disc
larval brain
salivary gland
S2 cells
f
X chromosome
Autosome
RNA levels relative to mle9/CyO-GFP ♂
roX1 roX2 socs16D Ucp4a CG5254 hh Pfk RpL32 ap vg so wg opa msl-2
mle⁹/CyO-GFP ♂
mle⁹/CyO-GFP ♀
mle⁹/mle⁹ ♀
g
protein levels
msl-2::3HA
line #7 #8 #9 control
kDa
RPB3
MSL2
HA
with tag
without tag
h
% eclosed flies (n=5)
msl-2::3HA
♂ males
♀ females
% of total flies eclosed / day
days 1 2 3 4 5 6 7 8
i
MSL2-HA ChIP
fold change over non-targets (normalized to Input)
Autosomal Promoters
Non-target
High Affinity Sites
vg ap so ent2 CG15011 roX1 roX2 socs16D CG4064
j
msl-2::3HA ♂
DAPI
MSL2 (HA)
MSL1
k
in vivo RNA interactome (FLASH)
MLE (S2 cells) ♂
MLE ♂
MLE ♀
MSL2-HA ♂
MSL2-HA ♀
untagged ♂
untagged ♀
roX1
roX2

Supplementary Figure 2

a) Genome browser snapshot comparing MSL2tg (male L3 larvae, this study), MSL2 (S2 cells[2]) and roX ChIRP (L3 larvae[3]) on X chromosome and autosomes. Data normalization is described in methods.

b) Heatmaps showing CLAMP (L3 larvae[4]) enrichment on HAS sorted by enrichment intensity. The HAS center was used as a reference point, while plotting the signal +/- 0.8Kb. The mean enrichment profile is shown on top of the heatmap, ChIP data normalization is described in methods.

c) Genome browser snapshot showing that MLE and MSL2tg spreading is pronounced at H3K36me3 positive regions, whereas H4K16ac spreads beyond active genes. roX2 ChIRP signal is confined to HAS. Data normalization is described in methods.

d) Analysis of X-linked peaks, which do not overlap with HAS. Mean enrichment profile at non-HAS peaks on the X in comparison to all autosomal sites is shown. The MEME motif analysis of these peaks is shown below, where the top-scoring motif was chosen.

e) Real-time RT-qPCR analyses of the indicated genes from the same samples shown in Figure 2g. The RNA level of each gene was calculated relative to *RpL32* expression as a reference gene. The barplot represents the average of 3 independent biological replicates with error bars indicating the SEM.

f) Real-time RT-qPCR analyses of the indicated genes from heterozygous *mle^9/CyO, GFP* (male, female) and homozygous *mle^9/mle^9* (female) L3 larvae. The RNA level of each gene was calculated relative to the geometric mean of *RpL32* and *Pfk* and expressed relative to the *mle^9/CyO, GFP* males. The barplot represents the average of 5 independent biological replicates with error bars indicating the SEM.

g) Characterization of fly lines, where the *msl-2* gene was CRISPR-tagged at its C-terminus with a 3HA-6His-Bio tag (*msl-2::3HA* line). Three independent lines were obtained (#7, #8, #9) and analyzed by Western blot from heads. They display identical protein expression levels compared to the endogenous MSL2 protein in wild-type Oregon R flies (lanes 7 and 8).

h) Male and female progeny of the homozygous *msl-2::3HA* line #7 in a total of 5 vials were counted each day after the first flies eclosed (*msl-2* null mutants are male-specific lethal, whereas females are unaffected). The barchart represents the average of 5 vials (progeny eclosed on each day in % of the total number of flies eclosed per

vial) and error bars represent the SD. *msl-2::3HA* males and females display equal viability and no developmental delay.

i) ChIP-qPCR analyses of endogenously tagged *msl-2::3HA* male L3 larvae. The barplot shows the average of 3 independent biological replicates / experiments, the error bars represent the SEM. Enrichment values were calculated relative to input and serial dilutions performed to account for primer efficiency. The data is expressed as fold change enrichment over the non-targets.

j) Polytene squash of the endogenous *msl-2::3HA* line. Squashes were immunostained with MSL1 and HA antibodies, respectively. The transgene displays the expected localization to the X chromosome. Scale bar = 50 µm.

k) Genome browser snapshots showing MLE and MSL2-HA binding to roX1 and roX2 RNA in male and female L3 larvae. Oregon R L3 larvae (untagged) were used as controls. Note that in endogenously tagged *msl-2::3HA* line, females do not express MSL2-HA protein. The data range represents the number of uniquely mapped alignments for each profile. Tracks show merged biological duplicates except for the untagged larvae.

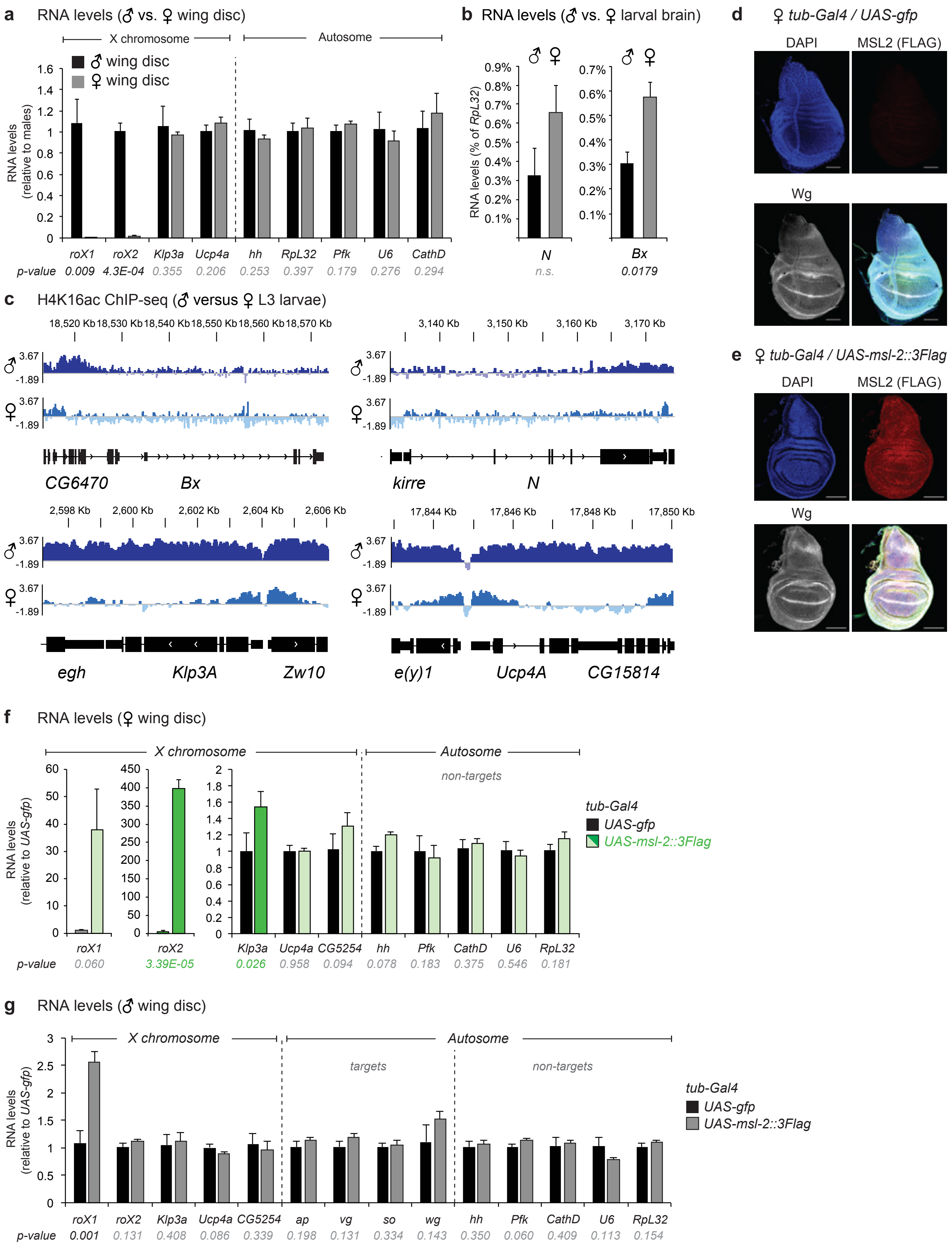
a RNA levels (♂ vs. ♀ wing disc)
X chromosome
Autosome
♂ wing disc
♀ wing disc
RNA levels (relative to males)
roX1 roX2 Klp3a Ucp4a hh RpL32 Pfk U6 CathD
p-value 0.009 4.3E-04 0.355 0.206 0.253 0.397 0.179 0.276 0.294
b RNA levels (♂ vs. ♀ larval brain)
RNA levels (% of RpL32)
N n.s.
Bx 0.0179
c H4K16ac ChIP-seq (♂ versus ♀ L3 larvae)
CG6470 Bx
kirre N
egh Klp3A Zw10
e(y)1 Ucp4A CG15814
d ♀ tub-Gal4 / UAS-gfp
DAPI
MSL2 (FLAG)
Wg
e ♀ tub-Gal4 / UAS-msl-2::3Flag
DAPI
MSL2 (FLAG)
Wg
f RNA levels (♀ wing disc)
X chromosome
Autosome
non-targets
RNA levels (relative to UAS-gfp)
tub-Gal4
UAS-gfp
UAS-msl-2::3Flag
roX1 roX2 Klp3a Ucp4a CG5254 hh Pfk CathD U6 RpL32
p-value 0.060 3.39E-05 0.026 0.958 0.094 0.078 0.183 0.375 0.546 0.181
g RNA levels (♂ wing disc)
X chromosome
Autosome
targets
non-targets
RNA levels (relative to UAS-gfp)
tub-Gal4
UAS-gfp
UAS-msl-2::3Flag
roX1 roX2 Klp3a Ucp4a CG5254 ap vg so wg hh Pfk CathD U6 RpL32
p-value 0.001 0.131 0.408 0.086 0.339 0.198 0.131 0.334 0.143 0.350 0.060 0.409 0.113 0.154

Supplementary Figure 3

a) Real-time RT-qPCR analyses of the indicated genes in male and female wild-type Oregon R wing discs. The RNA level of each gene was calculated relative to the geometric mean of *RpL32*, *Pfk* and *U6* expression level and expressed relative to males. The bar plot represents the average of 4 independently collected samples each consisting of 2 wing discs with error bars indicating the SEM. P-values were calculated using a one-tailed t-test (males versus females).

b) Real-time RT-qPCR analyses of *N* and *Bx* in male and female wild-type L3 larval Oregon R brains. The barplot represents the average of 3 independently collected samples each consisting of 1 larval brain with error bars indicating the SEM, P-values were calculated using a one-tailed t-test (males versus females).

c) Genome browser snapshot showing H4K16ac enrichment in wild-type male and female L3 larvae on the X-linked *Bx*, *N*, *Klp3a* and *Ucp4a*. Data normalization is described in methods.

d)-e) Immunostainings of female wing discs with FLAG (red), Wingless (Wg, white) and DAPI in blue. The genotype in d) was *w ;; tub-Gal4 / UAS-gfp* and in e) *w ;; tub-Gal4 / UAS-msl-2::3Flag*. Scale bar = 50 µM.

f) As in a), the data is expressed relative to the *UAS-GFP* expressing control samples. P-values were calculated using a one-tailed t-test (*UAS-msl-2::3Flag* flies versus *UAS-gfp* controls). The genotype of the female flies was *w ;; tub-Gal4 / UAS-msl-2::3Flag* or *w ;; tub-Gal4 / UAS-gfp.*

g) As in f), but for males *w / Y ;; tub-Gal4 / UAS-msl-2::3Flag* or *w / Y ;; tub-Gal4 / UAS-gfp* flies.

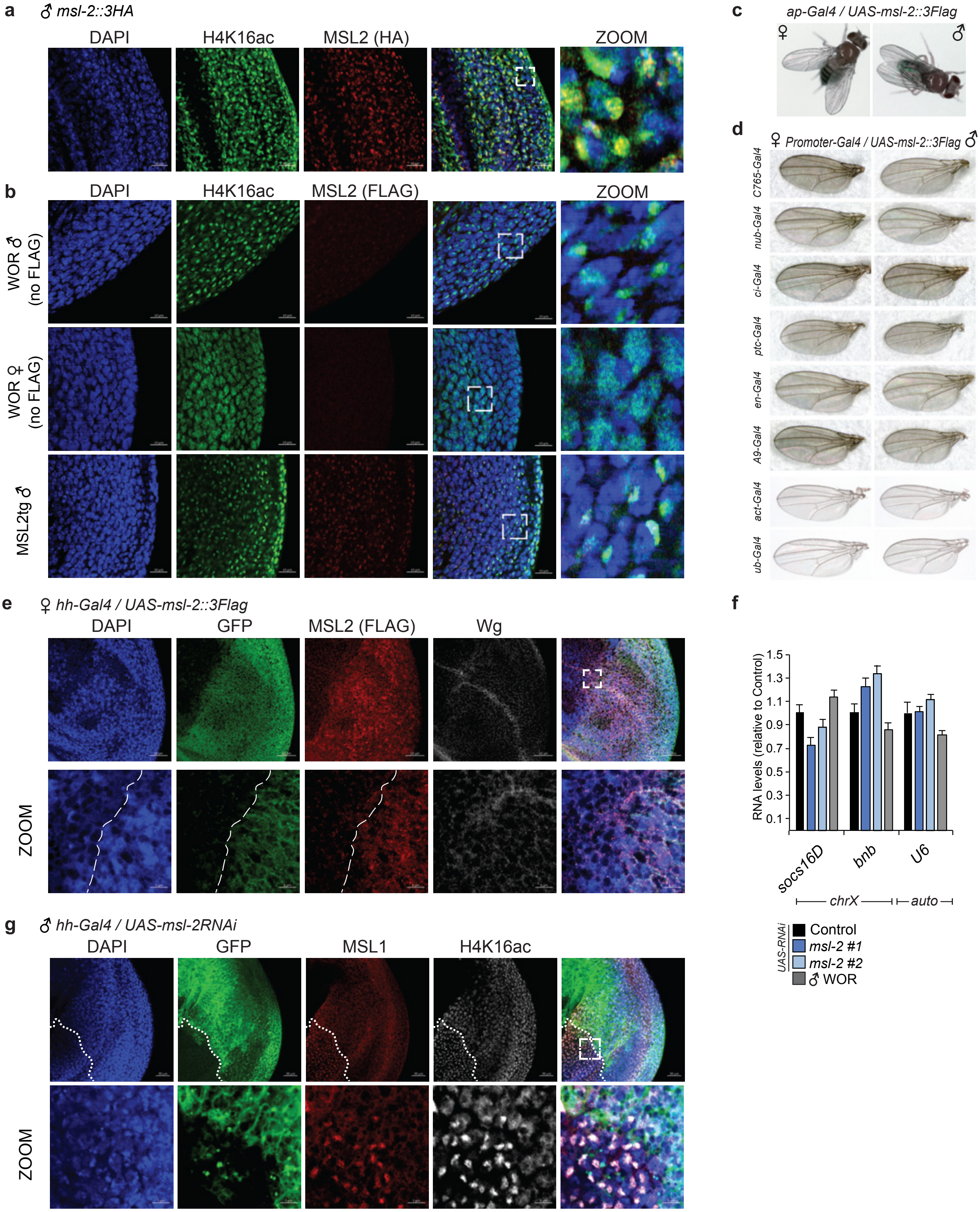
a
♂ msl-2::3HA
DAPI
H4K16ac
MSL2 (HA)
ZOOM
b
DAPI
H4K16ac
MSL2 (FLAG)
ZOOM
WOR ♂ (no FLAG)
WOR ♀ (no FLAG)
MSL2tg ♂
c
ap-Gal4 / UAS-msl-2::3Flag
♀
♂
d
♀ Promoter-Gal4 / UAS-msl-2::3Flag ♂
C765-Gal4
nub-Gal4
ci-Gal4
ptc-Gal4
en-Gal4
A9-Gal4
act-Gal4
ub-Gal4
e
♀ hh-Gal4 / UAS-msl-2::3Flag
DAPI
GFP
MSL2 (FLAG)
Wg
ZOOM
f
RNA levels (relative to Control)
1.5
1.3
1.1
0.9
0.7
0.5
0.3
0.1
socs16D
bnb
U6
chrX
auto
UAS-RNAi
Control
msl-2 #1
msl-2 #2
♂ WOR
g
♂ hh-Gal4 / UAS-msl-2RNAi
DAPI
GFP
MSL1
H4K16ac
ZOOM

Supplementary Figure 4

a) Immunostainings of male *msl-2::3HA* wing discs with H4K16ac (green), HA (red), and DAPI in blue. The very right panel shows a zoom from the merged panel. Scale bar = 10 μM. The signal for MSL2-HA and H4K16ac overlap in the X chromosomal territory within the male nucleus.

b) Immunostainings of wing discs with H4K16ac (green), FLAG (red) and DAPI (blue). WOR refers to wild-type Oregon R, MSLtg to *msl-2*^{227} / *msl-2*^{km}; *tub-Gal4 / UAS-msl-2::3Flag* wing discs. The very right panel shows a zoom from the merged panel. Scale bar = 10 μM.

c) Pictures of female and male adult flies expressing *ap-Gal4 / UAS-msl-2::3Flag* (BL3041).

d) Pictures of wings of female and male adult flies expressing *UAS-msl-2::3Flag* using the indicated Gal4-drivers (see methods).

e) Immunostainings of female wing discs (*UAS-gfp / w ;; hh-Gal4 / UAS-msl-2::3Flag*) with GFP (green), FLAG (red), Wingless (Wg, white) and DAPI (blue). The bottom panel shows a zoom from the top panel. Scale bar = 5 μM.

f) As in Figure 4f) Real-time RT-qPCR analyses of the indicated genes in male wing discs upon *UAS-msl-2*^{RNAi} with *hh-Gal4*.

g) Immunostainings of male wing discs (*UAS-gfp / Y ;; hh-Gal4 / UAS-msl-2*^{RNAi}, BDSC 31627) with GFP (green), MSL1 (red), H4K16ac (white) and DAPI (blue). The bottom panel shows a zoom, DAPI is shown in blue. Scale bar = 5 μM.

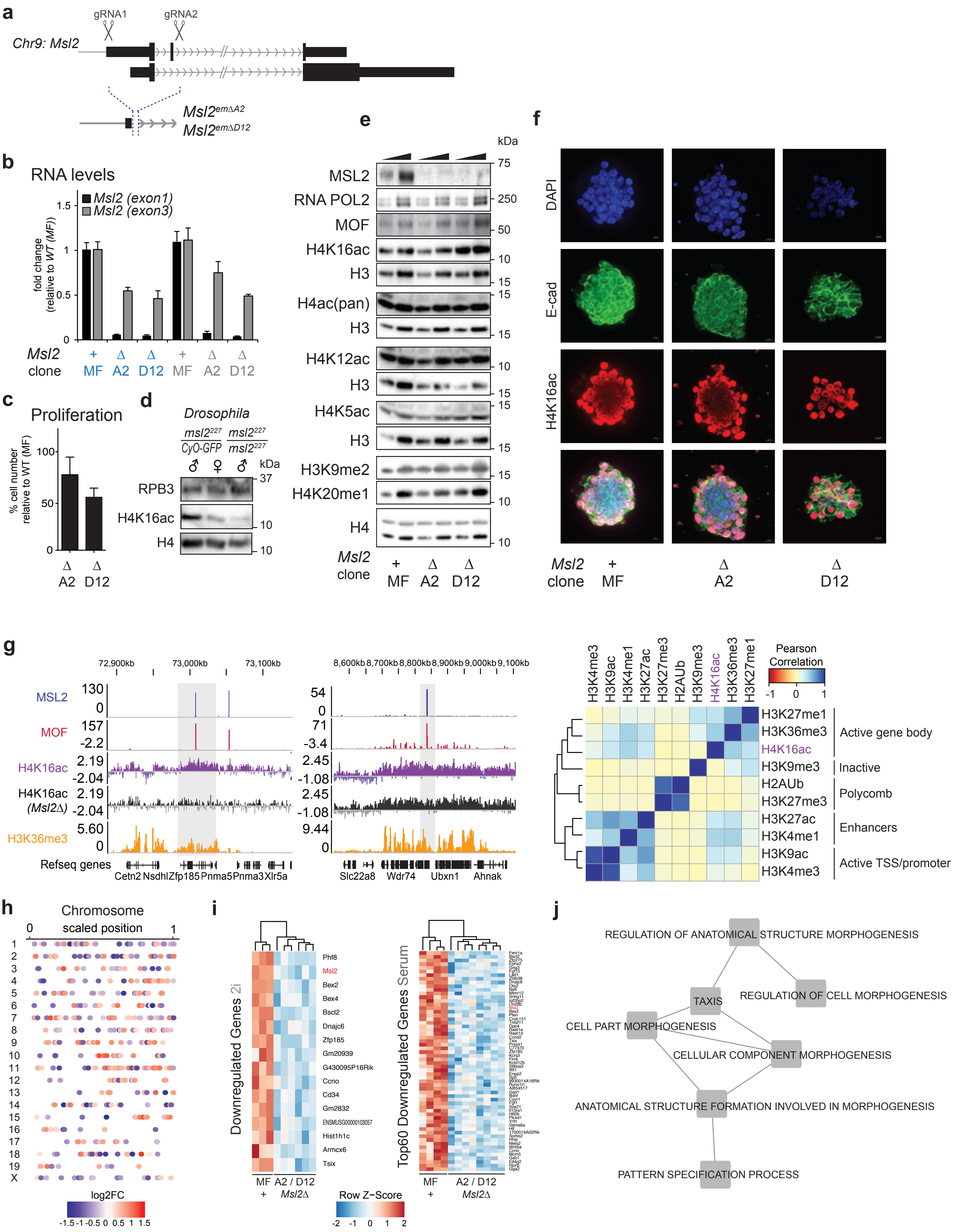

a
gRNA1
gRNA2
Chr9: Msl2
Msl2emΔA2
Msl2emΔD12
b
RNA levels
Msl2 (exon1)
Msl2 (exon3)
fold change (relative to WT (MF))
Msl2 + Δ Δ + Δ Δ
clone MF A2 D12 MF A2 D12
c
Proliferation
% cell number relative to WT (MF)
Δ Δ
A2 D12
d
Drosophila
msl2227 / CyO-GFP
msl2227 / msl2227
kDa
RPB3
H4K16ac
H4
e
kDa
MSL2
RNA POL2
MOF
H4K16ac
H3
H4ac(pan)
H3
H4K12ac
H3
H4K5ac
H3
H3K9me2
H4K20me1
H4
Msl2 clone + MF Δ A2 Δ D12
f
DAPI
E-cad
H4K16ac
Msl2 clone + MF Δ A2 Δ D12
g
MSL2
MOF
H4K16ac
H4K16ac (Msl2Δ)
H3K36me3
Refseq genes
Cetn2 Nsdhl Zfp185 Pnma5 Pnma3 Xlr5a
Slc22a8 Wdr74 Ubxn1 Ahnak
Pearson Correlation
H3K4me3 H3K9ac H3K4me1 H3K27ac H3K27me3 H2AUb H3K9me3 H4K16ac H3K36me3 H3K27me1
Active gene body
Inactive
Polycomb
Enhancers
Active TSS/promoter
h
Chromosome
scaled position
log2FC
i
Downregulated Genes 2i
Phf8
Msl2
Bex2
Bex4
Bscl2
Dnajc6
Zfp185
Gm20939
G430095P16Rik
Ccno
Cd34
Gm2832
Hist1h1c
Armcx6
Tsix
MF + A2 / D12 Msl2Δ
Top60 Downregulated Genes Serum
Row Z-Score
j
REGULATION OF ANATOMICAL STRUCTURE MORPHOGENESIS
TAXIS
REGULATION OF CELL MORPHOGENESIS
CELL PART MORPHOGENESIS
CELLULAR COMPONENT MORPHOGENESIS
ANATOMICAL STRUCTURE FORMATION INVOLVED IN MORPHOGENESIS
PATTERN SPECIFICATION PROCESS

Supplementary Figure 5

a) Scheme showing the CRISPR/Cas9-mediated strategy to delete *Msl2* in mESCs. The two independently obtained knock-out clones lack most of the 5'UTR, exon1 and exon2 affecting all isoforms.

b) Real-time RT-qPCR analyses of *Msl2* RNA levels in parental (MF clone) versus the two *Msl2* knock-out clones (A2 and D12) in Serum (blue, left) and 2i medium (grey, right). The RNA level was calculated relative to *Hprt*. The bar plot represents the average of 4 independent experiments with error bars indicating the SEM. Note that transcripts can still be detected from exon 3, which can be attributed to non-sense mediated decay (NMD).

c) Cell growth ratio of *Msl2*Δ cells (A2 and D12) versus parental MF cells after 4 days in culture. The barplot represents the average of 4 independent experiments with parental (MF) cells set to 100% and error bars indicating the SEM.

d) Cropped western blots showing bulk H4K16ac levels in heterozygous *msl-*2^{227}*/CyO, GFP* and *msl-*2^{227}*/msl-*2^{227} null mutant *Drosophila* L3 larvae. H4 and Rpb3 serve as loading controls.

e) Cropped western blots showing the impact of *Msl2* deletion on bulk levels of the indicated proteins and histone modifications in mESCs. The same extracts were analyzed on multiple gels / membranes. For each condition two different amounts were loaded.

f) Immunofluorescence of parental MF and *Msl2*Δ mESC colonies grown in 2i medium. Displayed stainings are DAPI (blue), E-cadherin (green) and H4K16ac (red). Scale bar = 10 µm.

g) Genome browser snapshot of MSL2, MOF, H4K16ac, H4K16ac (*Msl2*Δ) and H3K36me3 ChIP-seq at the *Zfp185* (left) and *Bscl2* (right) locus. The grey shaded area corresponds to approximately 50 kb. Normalization is described in methods. The right panel shows the Pearson correlation of several histone modifications compared to the H4K16ac ChIP-seq profiles generated in this study. Enrichment scores were calculated in 1kb tiled bins across the mouse genome, while excluding regions from the ENCODE blacklist. The Correlation was then plotted from the deeptools multibigwigSummary output using plotCorrelation function.

h) DE genes by RNA-seq of *Msl2*Δ cells in Serum were plotted according to their position on each chromosome, while scaling each chromosome to the same size. Each

dot represents a DE gene, where the color scale indicates the fold change of that particular gene.

i) Heatmaps of all downregulated genes in 2i (left) and the Top60 (by p-adj) downregulated genes in Serum grown mESCs (Parental MF versus *Msl2*Δ cells).

j) GO based gene set enrichment map of the DE genes upon *Msl2*Δ in Serum grown mESCs.

Supplementary Figure 1a

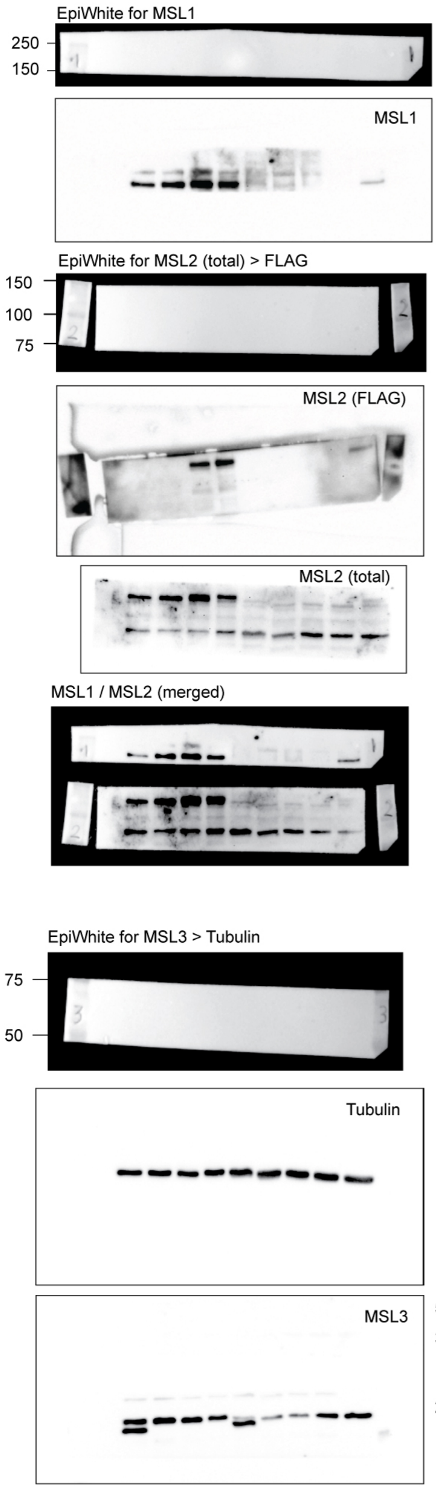

Supplementary Figure 5d

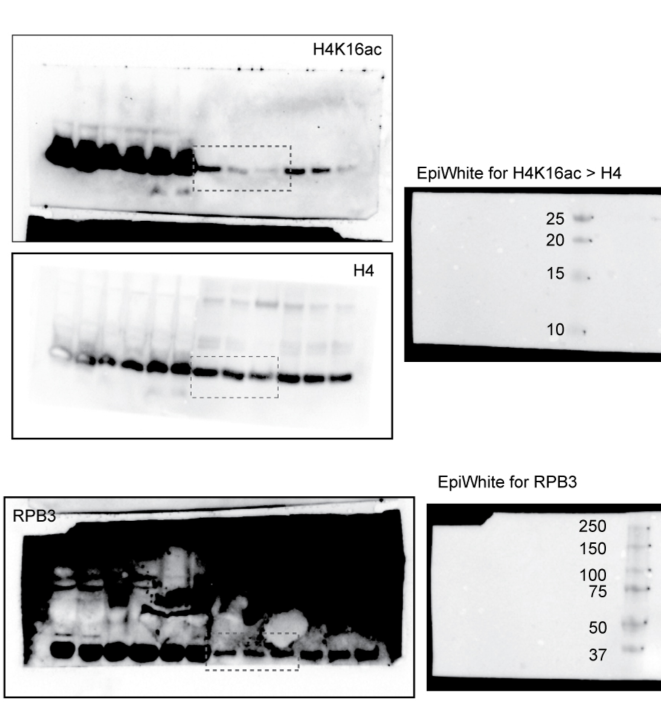

Supplementary Figure 2g

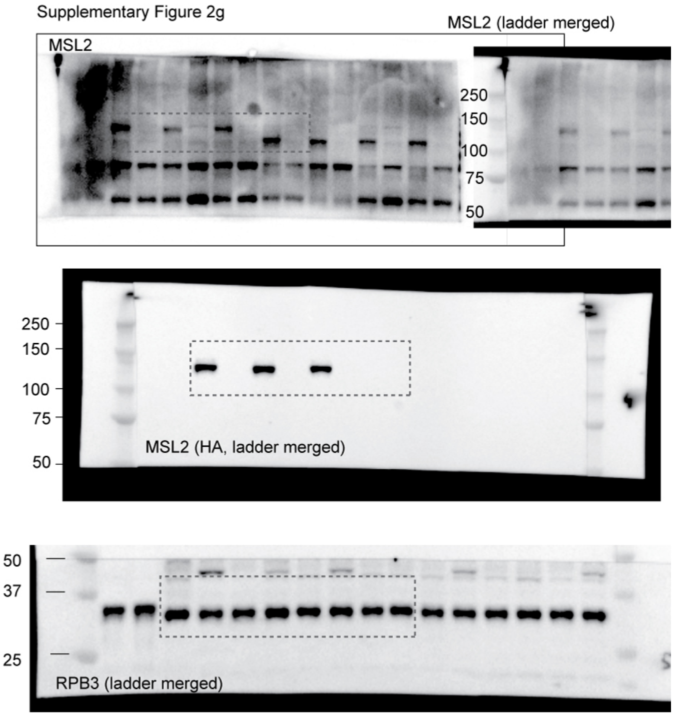

Supplementary Figure 5e

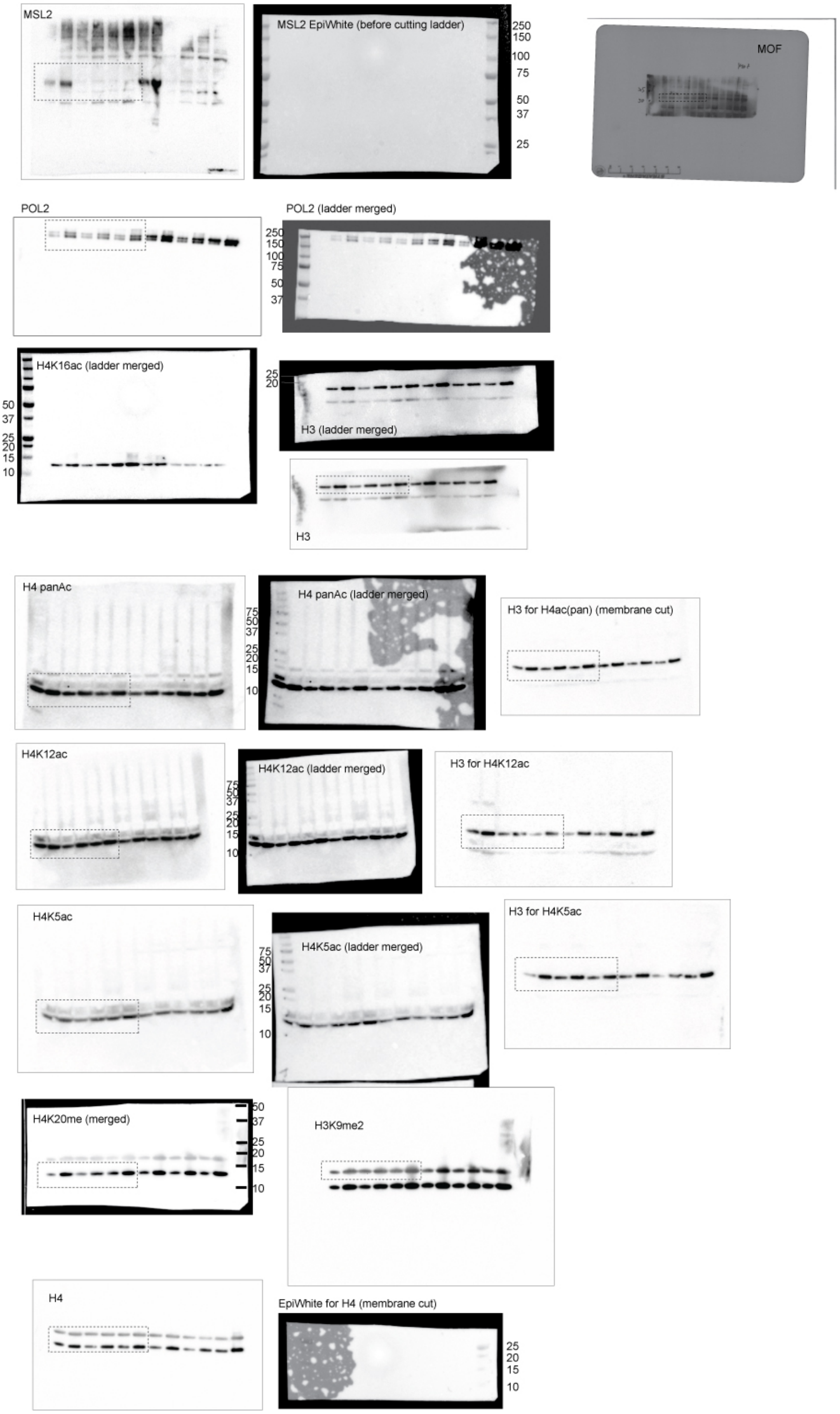

Supplementary Figure 6

Uncropped Western Blots with molecular weight markers. Note that for certain antibodies, the ladder causes background and had to be cut before applying HRP solution. Some blots are represented as merged with the molecular weight marker causing slightly different appearance of contrast than presented in the actual figures (blots marked as “ladder merged”). The molecular weight marker in Supplementary Fig. 5d and certain blots of Supplementary Fig. 5e was loaded together with sample due to limitation of wells. H3K9me2 and H4K20me1 antibodies were applied / detected at the same time.

RNA expression

ID	Sequence	Target	
**Drosophila**			
ck1313	TCAATCTCTAAGACAACGCCG	ap	fwd
ck1314	GCTATTGGACACTTGACACTGG	ap	rev
ck1315	CCATCGCTAAACCGCAAAAG	vg	fwd
ck1316	GTAGGCACCGTACATAACTTCG	vg	rev
ck1387	ATACTGCTTTGGGCAGGACC	wg	fwd
ck1388	CCAGCCCTGGTTACCGATTT	wg	rev
ck1317	GGAGATCGTGTTTTGAGCATG	hh	fwd
ck1318	AGGTTGCGGTCCATGAAG	hh	rev
ck1319	GCGCGTCCAGCTAAAATAAAG	Bx	fwd
ck1320	CTCCAACTCCAACTCCAACTC	Bx	rev
ck1321	ATTATAGCCCCACACTTTCCG	so	fwd
ck1322	ACACAGATCGATGCAGAAGTC	so	rev
ck640	ATCGGTTACGGATCGAACAA	RpL32	fwd
ck641	GACAATCTCCTTGCGCTTCT	RpL32	rev
ck439	GCTTCGGCAGAACATATACT	snRNA::U6	fwd
ck440	ACGATTTTGCGTGTCATCCT	snRNA::U6	rev
ck838	AAGGGCTATGAGGGCAGAGA	N	fwd
ck839	AGTCACCGATCCCATCCAGA	N	rev
ck447	GCCATCGAAAGGGTAAATTG	rox2	fwd
ck448	CTTGCTTGATTTTGCTTCGG	rox2	rev
ck449	TCCCACCCGAATAACCAACC	rox1	fwd
ck450	GCATAGGCTTTCAATACCGTTCC	rox1	rev
ck1247	GCCCAGACGGCATACTTGAA	msl-2	fwd
ck1248	CCCGCCGTTTGGAAAGATTC	msl-2	rev
ck644	CTGAGGGCAAGTTCAAGGAG	Pfk	fwd
ck645	AAGCCACCAATGATCAGGAG	Pfk	rev
ck451	CGCAAGGAGTTCACACAGAA	Ucp4a	fwd
ck452	CTCCATTTGGATTTGCACCT	Ucp4a	rev
ck445	CATTCCCATTCGGAGGAGTA	Klp3a	fwd
ck446	GCAGCTCCTGTTTGAGATCC	Klp3a	rev
OAG456	ATCCTAGGCCTGGGCTACAA	CathD	fwd
OAG457	AGAATGAGAACACCGGAGCG	CathD	rev
gs33f	CCAGCAAGGTGGTCAAGAAG	Rpl22	fwd
gs33r	CCATGATGCTATCCTCAGCA	Rpl22	rev
ck642	CTCCTACTGGAAGGGCATCA	CG5254	fwd
ck643	CCAGCCAGAGAAAAGGTCAG	CG5254	rev
ck1480	gatacaatttggtacagtgaaatatgg	salm	fwd
ck1481	ctgatcgctaccgatgtcttt	salm	rev
ck1474	atcgagatggccttgctg	ssp4	fwd
ck1475	catggtctcccacttcatca	ssp4	rev
ck1476	gtttggctaaatcccaagga	esn	fwd
ck1477	cttcctcctgctcttgtcca	esn	rev
ck1468	cgtcggacaagccctaca	opa	fwd
ck1469	gctcttctcgtccacattgc	opa	rev
ck1478	agacgaatgcaatccagga	socs16D	fwd
ck1479	gaccccagtaccagccatag	socs16D	rev
131a	tcaactacctagtgcgcgtg	mle	fwd
131b	tcaaacactcgcttctgctg	mle	rev
ck834	TCCAACCAGTGTAGCATCCA	roX2 intron	fwd
ck835	AGGATTGTCATAGGCGCAAC	roX2 intron	rev
**Mouse**			
	AACCCCGTGAATGCTACTG	mmMsl2 (exon1)	fwd
	CTGTCGGAAGTAAGGCAAGAG	mmMsl2 (exon1)	rev
	GGTTATCATGTACAGCAGCAACTC	mmMsl2 (exon3)	fwd
	GTAGAAATTGGAAGTGGCTGAACT	mmMsl2 (exon3)	rev
	GTATACCTAATCATTATGCCGAGGA	mmHprt	fwd
	GACATCTCGAGCAAGTCTTTCA	mmHprt	rev
	GGCAGAACTTTTGACAGCTC	mmPhf8	fwd
	TTCAAGACAAGGATAGGCACG	mmPhf8	rev
	CAGATTGACTGGAAACCGAGAG	mmBex2	fwd
	CACGCCTTGTTCCACTTTG	mmBex2	rev
	GGGTTGATGTGGTAGATGAGG	mmZfp185	fwd
	TGGGCAATCTTCTCGGTTG	mmZfp185	rev
	CCGACAAAGGGATAACTCACAC	mmBscl2	fwd
	AGGGCTCTCACCATCCTC	mmBscl2	rev
	GCAAAAGAGAAAGCCAAAGGG	mmLin28	fwd
	ACCACAGTTGTAGCATCTTGG	mmLin28	rev
	TTCCGGATTTTCACTCTGTCC	mmTsix	fwd
	GAGGGTTTGAGGGAGTGTG	mmTsix	rev

ChIP-qPCR

ID	Sequence	Target	
**Drosophila**			
ck988	TGCATTGGATTTACCGCTCCT	vg	fwd
ck989	GCCGCTCAATCGGAAGAGAA	vg	rev
ck992	ACGTGAATAAGGCAGCGGTA	ap	fwd
ck993	TGGCGCACAGCTTATACTCC	ap	rev
ck998	CGTCCCGCGCACAAAGTTAT	so	fwd
ck999	GCTTTTCTCTCGCTTCGTGTG	so	rev
ck923	ACCGCTCTCTTTCGGGACTTG	roX1 HAS	fwd
ck924	GGGTGAGTGAGACGGCCATAG	roX1 HAS	rev
ck927	GATTCGTGCCCAAAGTGAGGG	socs16D HAS	fwd
ck928	TCCCACCCACAACCAAAACCT	socs16D HAS	rev
ck1048	CGTATACGAGTCTTGAAAAGAAAG[illegible]	roX2 HAS	fwd
ck1049	CTCTCTAAGCCAGCACCGTT	roX2 HAS	rev
ck1050	GAGATAGCGATGGCGGTGTG	CG15767/CG4064 HAS	fwd
ck1051	CATGAAGCTTCCAACTATCTCGC	CG15767/CG4064 HAS	rev
ag382	CCTTTCGGAACTAGATCCCC	CG15011	fwd
ag383	AAGCCGGCGTTTTTGTCTAT	CG15011	rev
ag378	CGTAACGGCACCCCTCAA	Ent2	fwd
ag379	ACCGCACCGCACTACAAG	Ent2	rev
**Mouse**			
ck1108	TGTTGTGTTGCAAGTGTGGA	Zfp185	fwd
ck1109	AGTTGCCAGCACTGAGTACA	Zfp185	rev
ck1116	TTCCTGTGTCAGCTTGTCCC	Firre	fwd
ck1117	CCCCAGTAATGTCTTGCAGC	Firre	rev
ck1166	CCTCCTCTAGGCACGTAGTAGT	Bex2	fwd
ck1167	GGGATCCGATTGTGGCCC	Bex2	rev
ck1261	CTCGTTGGTTTGGCAGATG	Wap	fwd
ck1262	TCCATGTTCCCAAAAGCCAG	Wap	rev
ck1267	GCTACCCACTGTCAGGATC	Nanog	fwd
ck1268	TCTCCCTGCTCCCTCTTC	Nanog	rev
ck1158	CGGACTCGGTCCTTAGCAG	Arf1	fwd
ck1159	TCATCGTGGGAGTCAAGGGG	mmArf1	rev
	ctctaccacttggaccatatgac	Intergenic	fwd
	gggctccaaacagcatctcta	Intergenic	rev

Supplementary Table 1

List of qPCR primers used in this study

Supplementary References

1. Lam, K.C. et al. The NSL complex regulates housekeeping genes in Drosophila. *PLoS genetics* **8**, e1002736 (2012).
2. Straub, T., Zabel, A., Gilfillan, G.D., Feller, C. & Becker, P.B. Different chromatin interfaces of the Drosophila dosage compensation complex revealed by high-shear ChIP-seq. *Genome research* **23**, 473-485 (2013).
3. Quinn, J.J. et al. Rapid evolutionary turnover underlies conserved lncRNA-genome interactions. *Genes & development* **30**, 191-207 (2016).
4. Kuzu, G. et al. Expansion of GA Dinucleotide Repeats Increases the Density of CLAMP Binding Sites on the X-Chromosome to Promote Drosophila Dosage Compensation. *PLoS genetics* **12**, e1006120 (2016).
